# Development of Loop-Mediated Isothermal Amplification (LAMP) Assay for In-Field Detection of American Plum Line Pattern Virus

**DOI:** 10.3390/v16101572

**Published:** 2024-10-05

**Authors:** Slavica Matić, Arben Myrta

**Affiliations:** 1 Institute for Sustainable Plant Protection, National Research Council of Italy (IPSP-CNR), Strada delle Cacce 73, 10135 Turin, Italy; 2 Certis Belchim BV, Stadsplateau 16, 3521 AZ Utrecht, The Netherlands; arben.myrta@certisbelchim.com

**Keywords:** *Prunus serrulata* Lindl., American plum line pattern virus, sequencing, phylogenetic analyses, isothermal amplification, point-of-care detection, quarantine pathogen

## Abstract

American plum line pattern virus (APLPV) is the most infrequently reported Ilarvirus infecting stone fruit trees and is of sufficient severity to be classified as an EPPO quarantine A1 pathogen. In late spring, yellow line pattern symptoms were observed on leaves in a few flowering cherries (*Prunus serrulata* Lindl.) grown in a public garden in Northwest Italy. RNA extracts from twenty flowering cherries were submitted to Ilarvirus multiplex and APLPV-specific RT-PCR assays already reported or developed in this study. One flowering cherry (T22) with mixed prunus necrotic ringspot virus (PNRSV) and prune dwarf virus (PDV) infection also showed infection with APLPV. Blastn analysis of PCR products of the full coat protein (*CP*) and movement protein (*MP*) genes obtained from flowering cherry T22 showed 98.23% and 98.34% nucleotide identity with reference APLPV isolate NC_003453.1 from the USA. Then, a LAMP-specific assay was designed to facilitate the fast and low-cost identification of this virus either in the laboratory or directly in the field. The developed assay allowed not only the confirmation of APLPV (PSer22IT isolate) infection in the T22 flowering cherry but also the identification of APLPV in an asymptomatic flowering cherry tree (TL1). The LAMP assay successfully worked with crude flowering cherry extracts, obtained after manually shaking a single plant extract in the ELISA extraction buffer for 3–5 min. The developed rapid, specific and economic LAMP assay was able to detect APLPV using crude plant extracts rather that RNA preparation in less than 20 min, making it suitable for in-field detection. Moreover, the LAMP assay proved to be more sensitive in APLPV detection in flowering cherry compared to the specific one-step RT-PCR assay. The new LAMP assay will permit the estimation of APLPV geographic spread in the territory, paying particular attention to surrounding gardens and propagated flowering cherries in ornamental nurseries.

## 1. Introduction

American plum line pattern virus (APLPV), belonging to the *Bromoviridae* family (genus Ilarvirus), is the causal agent of plum line pattern disease [1]. Similar line pattern symptoms caused by this virus might be caused by prunus necrotic ringspot virus (PNRSV) and apple mosaic virus (ApMV), which contribute to a delay in its diagnosis, making it the least reported virus of the genus Ilarvirus [1]. Besides Japanese plum (*Prunus salicina* Lindl.), APLPV also infects other stone fruit trees such as plum (*Prunus domestica* L.), apricot (*Prunus armeniaca* L.), sweet cherry (*Prunus avium* L.), flowering cherry (*Prunus serrulata* Lindl.) and Yoshino cherry (Prunus × yedoensis Matsum.) [2,3,4,5,6,7].

APLPV virions are quasi-spherical particles with a size of 26–35 nm [8]. They contain three single-stranded positive RNAs with a total molecular size of 7.8 kb and a coat protein of 25 kDa [8,9]. There are limited sequences of several virus isolates in the GenBank database, and based on their sequences, the virus is characterized by low genetic diversity [10].

It was first reported in the USA on Japanese plum and flowering cherry [11,12]. Subsequently, it was found in New Zealand [13], Argentina [14], Palestine [8,15], Italy, Albania, Tunisia [3], Lebanon [5], Korea [6], Japan [16] and Australia [7] mainly with a limited presence. There are also reports of APLPV isolates from French and Canadian suppliers [17] or from India at GenBank database (Accession Number OQ513270), but with limited information on the precise geographic origin of the isolates or their plant hosts.

APLPV causes clear-cut line pattern disease with symptoms that vary depending on the host. Thus, on Japanese plum, a yellow pattern starts with chlorotic rings and an oak-leaf pattern and ends with yellow-to-ivory vein banding patterns. On flowering cherry, leaves have visible creamy, yellow-to-pink patterns in rings or oak-leaf forms. On peach, leaves may show yellow rings, lines, vein banding, and net or oak-leaf patterns [18]. The presentation of the virus is not highly specific because this virus can be found in a mixed infection with other Ilarviruses, i.e., prune dwarf virus (PDV), PNRSV and ApMV or apple chlorotic leaf spot trichovirus (ACLSV), the causal agents of similar line pattern symptoms on stone fruits [4,5,19].

APLPV belongs to the quarantine A1 pathogen of the European Plant Protection Organisation (EPPO). According to EPPO reports, APLPV is restricted in a few European and Mediterranean countries (Italy, Albania and Lebanon) with few presences, while it is not reported from other countries. However, it should be noted that there are no extended surveys for its presence as a quarantine pathogen. Meanwhile, it has very similar symptoms to other viruses and is also present in mixed infections as mentioned above. It has been reported sporadically in Italy during the last 20 years on Japanese plum in a couple of regions [3,20]. Moreover, it was reported also on a few flowering cherries in the Piedmont region [4]. The mode of virus transmission is still unknown, and the virus is not known to be spread by vectors. At the same time, there are reports of APLPV transmission by human work through grafting infected propagating material. The virus can also be transmitted by mechanical inoculation to experimental hosts such as cucumber, cowpea, *Nicotiana benthamiana*, *Chenopodium amaranticolor*, *Physalis pubescens* and *Petunia hybrida* [8,12,15].

The detection of APLPV is primarily based on molecular diagnostic techniques, i.e., PCR, including conventional PCR, multiplex PCR and real-time PCR [9,19,21,22]. Other detection methods such as dot blot hybridization [19] or ELISA [23] can also be applied. All of these techniques are time-consuming, laborious and expensive. They also require sample extraction and cannot be carried out directly in orchards. New innovative tools are needed to ensure the rapid, reliable, cheap and in-field detection of important plant pathogens, such as APLPV. One such technique is loop-mediated isothermal amplification (LAMP), which is increasingly used in the detection of plant viruses and is characterized by specificity, sensitivity, speed, simplicity and the possibility of being carried out in the field without the need for the laboratory extraction of nucleic acids from samples [23].

During late spring, yellow line pattern symptoms were observed again on leaves in a few flowering cherries in the province of Verbano-Cusio-Ossola (Piedmont, Northwest Italy), where APLPV was formerly found [4]. This study aimed to investigate the presence of APLPV in flowering cherries, and if confirmed, to study the genetic diversity of the virus isolate and to develop a LAMP assay for the rapid and specific detection of APLPV, which so far has not been developed for the detection of this virus.

## 2. Materials and Methods

### 2.1. Field Survey

A field survey was carried out in May (late spring) and September (late summer) observing twenty flowering cherries (*Prunus serrulata* Lindl.) in a public garden in Verbano-Cusio-Ossola province (Piedmont, Northwest Italy). In three of them, yellow line pattern symptoms were observed on leaves. Symptomatic and asymptomatic leaves of all inspected plants were taken and transported in a cold box to the laboratory.

### 2.2. RNA Extraction

Small pieces of three leaves, leaf petioles or twigs (100 mg) from each flowering cherry were used for total RNA extraction. Total RNA of twenty flowering cherries was isolated using an RNeasy Plant Mini kit (Qiagen, Hilden, Germany), according to the manufacturer’s instructions, and eluted in 50 μL of RNA-free water. The RNA concentration was measured using a NanoDrop 2000 Spectrophotometer (Thermo Fisher Scientific, Wilmington, DE, USA). The final RNA was adjusted to 20 ng/μL and then stored at −80 °C until use. In addition, RNAs were extracted from two flowering cherries named H1 and H2 grown at IPSP-CNR, Turin, which were not infected with Ilarviruses and were used as healthy controls.

### 2.3. One-Step RT-PCR

RNA extracts were subjected to three one-step RT-PCR assays specific for (a) Ilarviruses, (b) APLPV (previously developed) and (c) APLPV (developed in this study). 

(a) Ilarvirus multiplex one-step RT-PCR assay for the simultaneous detection of three Ilarviruses was performed [24] with slight modifications to amplify the PCR products of 517 bp (PDV), 417 bp (ApMV) and 356 bp (PNRSV). Reactions (25 μL) were prepared using a OneStep RT-PCR Kit (Qiagen, Hilden, Germany), following the manufacturer’s instructions, containing 20 ng of total RNA, 1× RT-PCR buffer, 0.6 μM of each primer (Ilarvirus-specific), 0.4 mM dNTPs and 1 μL of OneStep RT-PCR enzyme. One-step RT-PCR was conducted at 50 °C of reverse transcription for 30 min, with initial denaturation at 95 °C for 15 min, 35 cycles of 3-step cycling of 94 °C for 30 s, 52 °C for 45 s and 72 °C for 1 min, and final extension at 72 °C for 10 min. The RT-PCR products were electrophoresed using 1.5% agarose gel, stained with GelRed^®^ Nucleic Acid Gel Stain (Biotium, Hayward, CA, USA) and visualized using UV light. 

(b) The APLPV one-step RT-PCR assay was conducted as already described with minor modifications using only the set of primers specific for APLPV [19]. One-step RT-PCR reactions were prepared as described above except for using APLPV-specific primers (s, 5′-GGTCGTCAAGGGAGAGGC-3′ and as, 5′-GGCCCCTAAGGGTCATTTC-3′) to amplify a PCR product of 563 bp containing a partial coat protein (*CP*) gene and with an annealing temperature of 60 °C. 

(c) To confirm the presence of APLPV, two one-step RT-PCR assays were developed by designing specific primers based on the entire sequence of APLPV RNA3 (Accession Number NC_003453). APL_F1 (5′-GATTATCGTTCCGTCAGCCC-3′) and APL_R (5′-ATAGATGTCCTAGGGCCGAC-3′) primers were designed to amplify the full *CP* (651 bp) gene and its external region (1011 bp), while APL_s (5′-AGCTTGCTCCAGTGTTTGTG-3′) and APL_as (5′- AACCCACACTTTCCGCTCTA-3′) primers were designed for the amplification of the full movement protein (*MP*, 873 bp) gene and its adjacent region (1800 bp). The one-step RT-PCR reaction mix and conditions were as described above, applying 60 °C as the annealing temperature. 

### 2.4. Sequence Analyses

Both RT-PCR products of the T22 flowering cherry from the third PCR (c) were purified using a QIAquick PCR purification kit (Qiagen, Hilden, Germany), following the manufacturer’s instructions, and sequenced in both directions at the BMR Genomics. The obtained sequences of the APLPV isolate named PSer22IT were deposited in the NCBI GenBank database as accession numbers OP972867 (*CP*) and OP972868 (*MP*).

The BLAST algorithm “www.ncbi.nlm.nih.gov (accessed on 1 April 2024)” was used to compare the sequences of the PSer22IT isolate with the sequences of isolates available in the GenBank database. A multiple nucleotide (nt) and amino acid (aa) sequence alignment was performed using the CLUSTALW software (v. 2.1) [25], and the phylogenetic analyses were estimated using the maximum likelihood method with 1000 bootstrap replicates [26,27]. All analyses were conducted using the MEGA 11 (v. 11.0.13) software [28].

The role of natural selection in the evolution of APLPV isolates was studied by estimating the ratio of substitution rates at non-synonymous (dN) and synonymous sites (dS). These, together with other parameters of genetic diversity, were calculated using the DNA sequence polymorphism v.6 software [29]. Pairwise genetic identity calculations were carried out using MUSCLE alignments for both genes of APLPV using the SDT v1.4 software [30]. 

### 2.5. Design of LAMP Primers

Based on the APLPV PSer22IT genomic sequences (OP972868 and OP972867), two sets of primers were designed by the PrimerExplorer v.5 software (Eiken Chemical Corporation, Tokyo, Japan) (Table 1). The set of the LAMP primers designed within the *CP* gene contained 6 common primers for the LAMP assay, while the set of the LAMP primers within the *MP* gene contained 5 primers (lacking the loop LF primer). The low-complexity/secondary structure of the target *MP* sequence and algorithm features of the PrimerExplorer software (v. 5) resulted in a lack of the LF primer. However, the remaining 5 designed primers are sufficient for the efficient functioning of the LAMP assay. 

The specificity of primer sets was evaluated using the Nucleotide-BLAST algorithm “www.ncbi.nlm.nih.gov (accessed on 1 April 2024)” to assess the cross-reactivity with other viruses. This evaluation also included the testing of LAMP primers against the complementary genomes of viruses that infect flowering cherry: PDV (GenBank taxid:33760), prunus necrotic ringspot virus (PNRSV; GenBank taxid:37733), cherry virus A (CVA; GenBank taxid:42882), little cherry virus 1 (LChV-1; GenBank taxid:217686), little cherry virus 2 (LChV-2; GenBank taxid:154339), cherry necrotic rusty mottle virus (CNRMV; GenBank taxid:129143), cherry rasp leaf virus (CRLV; GenBank taxid:202566), cherry green ring mottle virus (CGRMV; GenBank taxid:65467), plum bark necrosis stem pitting-associated virus (PBNSPaV; GenBank taxid:675077) and tomato ringspot virus (ToRSV; GenBank taxid:12280).

### 2.6. APLPV LAMP Assay Optimization

The LAMP assay was optimized in a 12.5 µL reaction mixture that contained the following components: 0.5 µL of each inner prime (FIP and BIP), 0.05 µL of each outer primer (F3 and B3), 0.25 µL of each loop primer (LF and/or LB), 6.25 µL 2× Isothermal Mastermix ISO-004^®^ (OptiGene Ltd., Horsham, UK), 0.1 µL of AMV Reverse Transcriptase (Promega, Madison, USA), 2.5 ul of total RNA (undiluted or ten-fold serial dilutions until 10^−6^) and nuclease-free H_2_O to reach the final volume. The optimization of the LAMP assay was performed using RNA extracted from leaf samples of flowering cherry T22 from which the APLPV PSer22IT isolate was previously obtained by one-step RT-PCR. As a healthy control, RNA extracted from leaves of flowering cherry H1, free of Ilarvirus infection, was used.

The LAMP assay was carried out at 60, 63 and 65 °C (according to OptiGene instructions) for 60 min, and fluorescence was measured every 60 s, using the CFX96 Real-Time PCR Detection system (Bio-Rad, Hercules, CA, USA). Melting curves for LAMP analysis were calculated between 60 and 95 °C (ramp rate of 0.05 °C/s, plate readout every 15 s).

### 2.7. In-Field LAMP Assay

During May, the LAMP assay was tested directly in the field in a public garden in Verbania where APLPV infection was found. Since the flowering cherry tree from which the PSer22IT isolate originated was a dying tree at the time, we tried to collect samples of the remaining roots from it. From the same location, leaf samples were taken from additional 19 flowering cherry trees that did not show typical symptoms of Ilarvirus infection. Collected roots and leaves were used to prepare crude extracts as described by Matic et al. [31]. Briefly, leaf or root tissue (100 mg) of each sample was placed in 5 mL tubes containing one tungsten bead (OptiGene, Horsham, UK) and a 500 µL extraction buffer, either Tris-EDTA-Triton X-100 (TET) buffer (20 mM Tris-HCl pH 8, 20 mM EDTA, 1% Triton X-100) [32] or ELISA extraction buffer (1× phosphate-buffered saline, pH 8.2; 2% polyvinylpyrrolidone MW 24,000, 1% PEG MW 6000, 0.05% Tween 20). Samples were manually shaken for 3–5 min, and then the homogenate was diluted 1:4. In total, 2.5 μL of 1:4 diluted crude extracts were added to 10 μL of the LAMP reaction mix, which contained AMV Reverse Transcriptase, as described in paragraph 2.6.

RNA extracts containing APLPV PSer22IT isolate and RNA extracts obtained from leaves of healthy flowering cherry (H1), not infected with Ilarviruses, previously prepared in the laboratory, were used as positive and negative controls. The portable bCube2 instrument (Hyris, London, UK) supplied with a battery was used to perform the LAMP assay in the field, applying the amplification temperature of 65 °C for 60 min.

The remaining parts of the leaf and root samples used in the in-field LAMP assay were transported in a cold box containing ice packs to the laboratory, where they were used for RNA extraction as described in paragraph 2.2. These RNA samples were used at 10^−2^ dilution for an APLPV-specific one-step RT-PCR assay to confirm the results of the in-field LAMP assay.

## 3. Results

### 3.1. Field Survey

**Figure 1:**
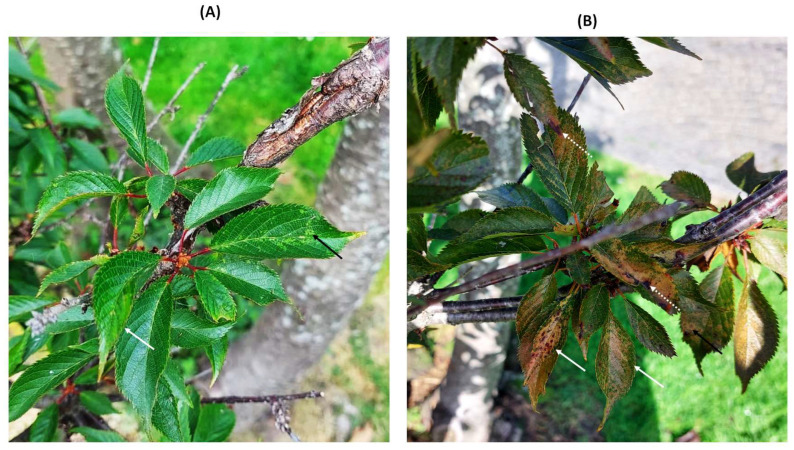
Symptoms of yellow line pattern on leaves on flowering cherry (*Prunus serrulata* Lindl.) cultivated in a public garden in Verbano-Cusio-Ossola province (Piedmont, Northwest Italy). Symptoms during May (**A**) and September (**B**) refer to the T22 tree from which the APLPV PSer22IT isolate was obtained. White arrows point to the yellow line pattern, black arrows to chlorotic rings, and dashed white arrows to necrotic leaf parts.

Out of twenty inspected flowering cherries in a public garden in Verbano-Cusio-Ossola province, a yellow line pattern with chlorotic ring symptoms resembling APLPV and other Ilarviruses infection was observed in three plants. The line pattern was observed sporadically on some branches of three flowering cherry trees. It gradually changed color from yellow during spring to orange and light brown at the end of summer (Figure 1).

### 3.2. One-Step RT-PCR

**Figure 2:**
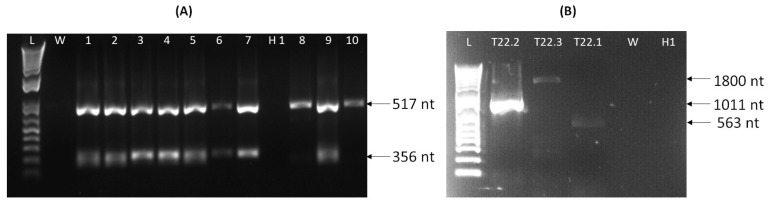
Agarose gel electrophoresis of multiplex Ilarvirus RT-PCR amplicons (**A**) and APLPV-specific RT-PCR amplicons (**B**) obtained during the field survey. (**A**) A one-step RT-PCR assay for the simultaneous detection of three Ilarviruses [24] was performed to amplify PCR products of 517 bp (PDV) and 356 bp (PNRSV). (B) One-step RT-PCR assays for specific APLPV detection to amplify the entire *CP* gene and its external regions (1011 bp, this study), the entire movement protein (*MP*) gene with adjacent genomic regions (1800 bp, this study) and the partial coat protein (*CP*) gene (563 bp, the assay described by Sánchez-Navarro et al [19]) from naturally infected flowering cherry tree (T22). L, 1kb plus bp DNA ladder; W, water control; Lanes 1–6, samples from different tissue materials of flowering cherry T22 infected by PNRSV, PDV and APLPV; Lane 7, flowering cherry T23 infected by PNRSV and PDV; Lane 8, flowering cherry T25 infected by PDV; Lane 9, flowering cherry T24 infected by PNRSV and PDV; Lane 10, flowering cherry T26 infected by PDV; H1, healthy flowering cherry control (grown at IPSP-CNR, Turin) that was not infected with Ilarviruses; the samples in lines 1, 2, 7, 8, 9, 10, and H1 correspond to leaf samples, while lines 3–4 represent leaf petiole samples, and lines 5–6, twig samples. Lanes T22.1, T22.2 and T22.3—flowering cherry T22.

Five of twenty samples gave positive results by Ilarvirus multiplex one-step RT-PCR. Within these five samples, two samples were asymptomatic in the field survey and showed infection with PDV, while the three remaining symptomatic samples with yellow patterns had mixed infection of PNRSV and PDV (Figure 2A). One sample from the T22 tree with mixed PNRSV and PDV infection resulted to also be infected with APLPV using the virus-specific RT-PCR assay [19] (Supplementary Figure S1). 

The infection of this sample with APLPV was confirmed by the one-step RT-PCR assays developed in this study (Figure 2B). Both developed RT-PCR assays showed specific APLPV amplification using the designed APL_F1 and APL_R primers (1011 bp) within the *CP* gene and its external region, and the APL_s and APL_as primers (1800 bp) within the *MP* gene and its nearby region. Neither set of primers observed non-specific reaction in the healthy and water control samples. The one-step RT-PCR reaction mix and conditions were used for both assays as described above, applying 60 °C as annealing temperature. 

Obtained amplicons (APL_F1 × R, and APL_s × as) with the developed one-step RT-PCR assays were sequenced, and the complete sequences of the *CP* and *MP* genes from the APLPV isolate, designated here as PSer22IT, were obtained.

### 3.3. Sequence Analyses

Blastn analysis of PSer22IT sequences showed 98.23% and 98.34% nucleotide identity and 98.62% and 98.88% amino acid identity with reference APLPV isolate NC_003453.1 from Japanese plum from the USA in the *CP* and *MP* genomic regions, respectively. Compared to other APLPV isolates, the PSer22IT showed the highest similarity with American PlmUs.125 isolate from Japanese plum in both genes (98.93% in *CP* and 98.75% in *MP*). Moreover, it displayed the highest amino acid identity with Indian PM1 isolate from plum in CP (99.54%) and with Albanian PlmAl.del isolate from Japanese plum (99.25%) in MP. 

**Figure 3:**
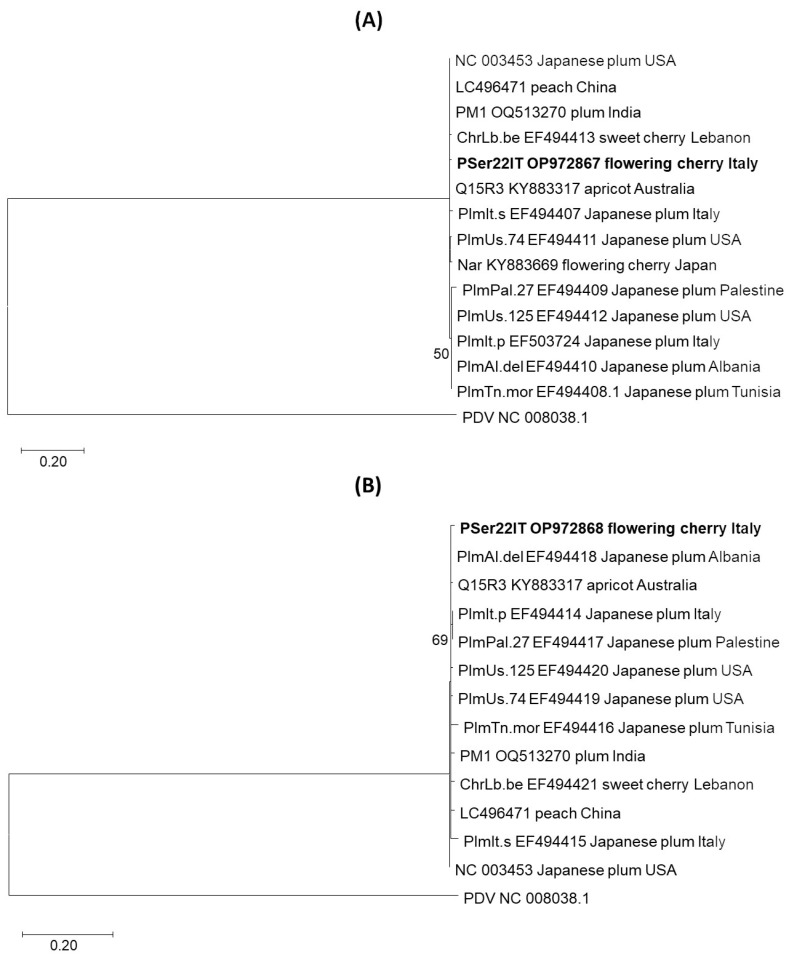
Phylogenetic tree of APLPV isolates based on amino acid sequences of (**A**) CP and (**B**) MP, inferred from a maximum likelihood analysis. The PSer22IT isolate (in bold) and thirteen reference isolates were included in the analysis. The isolate name (when known), accession number, the host and the origin of each isolate are shown. The bootstrap consensus tree of 1000 replicates and the bootstrap values above 50% are presented. Prune dwarf virus NC_008038.1 was used as an outgroup.

Phylogenetic analyses of the PSer22IT isolate and all available isolates from the GenBank database based on CP and MP amino acid sequences confirmed its assignment to APLPV. The maximum likelihood phylogenetic CP and MP trees grouped all APLPV isolates into one cluster including the PSer22IT and all reference isolates from 11 countries and six plant host species in both phylogenetic analyses (Figure 3). No grouping of isolates based on geographic origin or plant host was observed in either gene.

The pairwise genetic identity of sequences of all available APLPV isolates from the GenBank database was carried out, and the Italian sequences showed the identity to be higher than 98% in both genes (*CP* and *MP*) (Supplementary Figure S2). These sequences originated from two hosts and three geographical regions: Japanese plum (Apulia and Sicily) and flowering cherry (Piedmont). Moreover, the sequences of Japanese plum isolates had higher identity among them compared to the PSer22IT sequence originating from the other host (flowering cherry) in both genes: 99 vs. 98%. 

Synonymous substitutions (dS) (*CP*: 0.04196 and *MP*: 0.05152) were higher in APLPV isolates than the nonsynonymous substitutions (*CP*: 0.00387 and *MP*: 0.00568) per site, resulting in a dNS/dS ratio of 0.092 (*CP*) and 0.110 (*MP*). Furthermore, a low degree of overall nucleotide diversity (π) was determined (*CP*: 0.01406 and *MP*: 0.01613) (Table 2). Tajima’s D, Fu’s and Li’s D and Fu’s and Li’s F tests were negative, which may indicate the occurrence of selective processes or population expansion.

### 3.4. LAMP Primers

**Figure 4:**
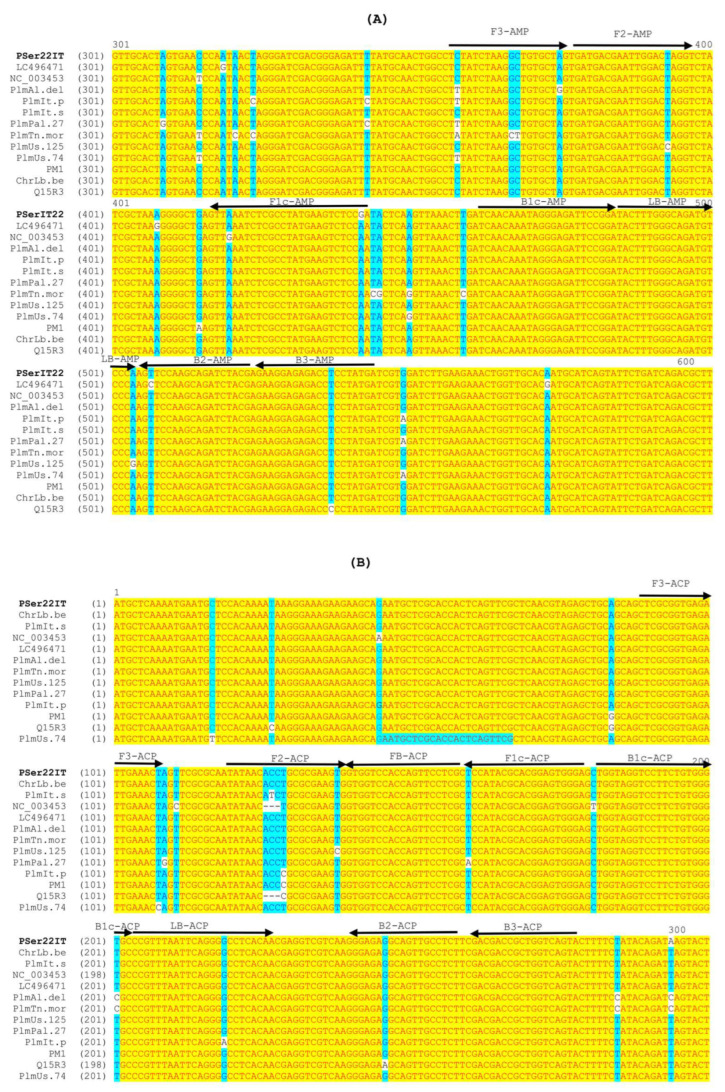
Schematic representation of LAMP primers designed in APLPV movement protein (*MP*) gene (**A**) and coat protein (*CP*) gene (**B**) for specific virus detection. All primers are indicated by arrows above the sequence alignment of a portion of the respective gene (MP in panel **A** and CP in panel **B**) from the APLPV isolates available in GenBank, including the APLPV PSer22IT isolate from this study. F3 and B3 are external primers, and FB and LB are loop primers. F1c and F2 portions form FIP, and B1c and B2 fragments form BIP.

Two sets of the LAMP primers were designed and tested with the studied isolates (Table 1). Both sets were supposed to be specific not only with APLPV isolates from this study but also with isolates from other geographic areas and countries considering the low genetic variability among aligned sequences of viral isolates found worldwide (Figure 4). The first set of primers designed on the *MP* region showed more specific results and the rapid detection of the APLPV PSer22IT isolate in flowering cherry T22. The second set of primers designed in the *CP* gene showed non-specific melting curves in healthy samples and water control, and slower amplification (24–35 min) in APLPV-infected samples of the T22 flowering cherry compared to the first set of primers (Supplementary Figure S3). The first set of primers did not give a specific reaction in samples infected with other Prunus infecting viruses and the healthy control, and based on all the results obtained, the first set of primers was chosen for further experiments. 

### 3.5. LAMP Assay

**Figure 5:**
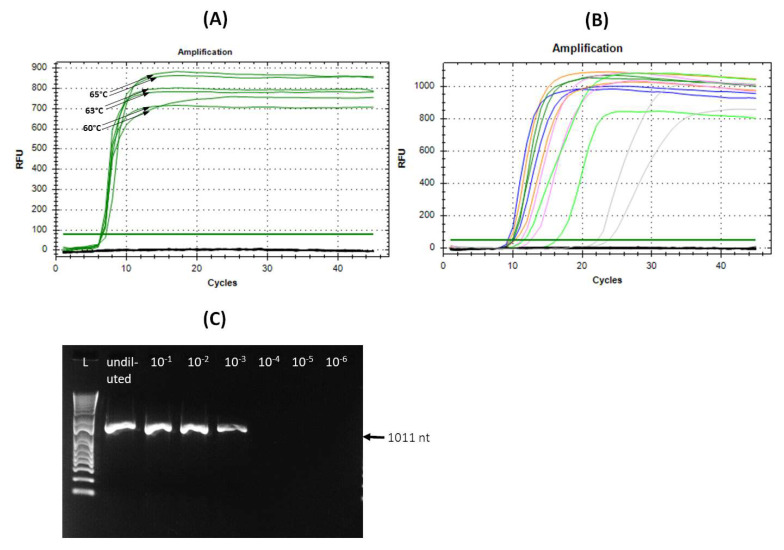
The optimization of real-time LAMP assay using different amplification temperatures (**A**) and diluted RNA extracts (**B**), and its comparison with the one-step RT PCR assay developed in this study (**C**). Both assays, LAMP and one-step RT-PCR, were performed using RNA extracted from leaf samples of flowering cherry T22, undiluted or at dilutions (10^−1^–10^−6^). The three amplification temperatures (60, 63 and 65° C) are shown with arrows in panel A, while the amplification curves obtained from undiluted RNA and diluted RNA are shown in panel B in the following colors: blue = undiluted, 10^−1^ = orange, 10^−2^ = green, 10^−3^ = pink, 10^−4^ = light green and 10^−5^ = gray. Unamplified signals from healthy control in panels A and B and APLPV- infected RNA (diluted 10^−6^) in panel B are shown in black. As a healthy control, RNA extracted from leaves of flowering cherry H1, free of Ilarvirus infection, was used. The agarose gel electrophoresis of the one-step RT-PCR performed using APL_F1 x R primers is shown in panel C. L–1kb plus bp DNA ladder.

The developed LAMP assay with selected MP primers worked best at 65 °C (Figure 5A). It was highly sensitive, permitting us to amplify APLPV up to 10^−5^ dilution, which corresponded to 0.5 pg of the total RNA per µL of the reaction mix in less than 30 min (Figure 5B). Through comparing all dilutions, a 10^−2^ dilution gave the best results with a short amplification range (around 10 min) and good technical reproducibility (Figure 5B). When the LAMP assay was compared with the one-step RT-PCR assay developed in this study using the same RNA extract at the same dilutions, it proved to be more sensitive because the APLPV-specific RT-PCR assay using APL_F1 × R primers gave positive results only up to a 10^−3^ RNA dilution (Figure 5C).

**Figure 6:**
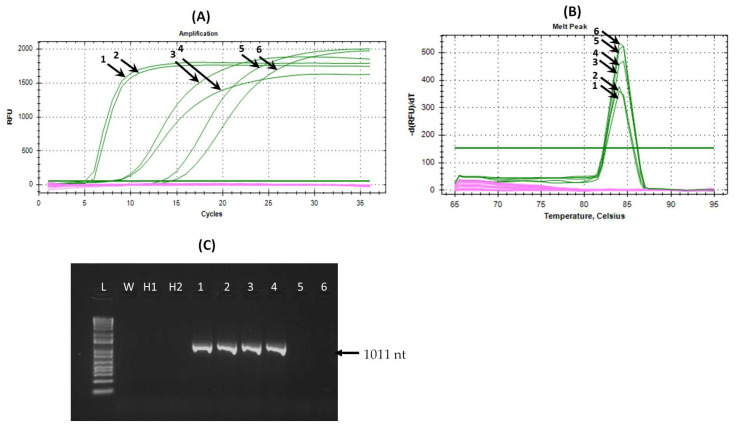
The laboratory detection of APLPV in flowering cherry by real-time LAMP and one-step RT-PCR (*CP*) assays developed in this study. The results are visualized as follows: (**A**) LAMP; (**B**) LAMP melting curve; (**C**) agarose gel results of the one-step RT-PCR. APLPV-infected samples from the T22 flowering cherry are indicated in green (each sample with the same color gradation). Healthy control samples (flowering cherry trees named H1 and H2 grown at IPSP-CNR, Turin, that were not infected with Ilarviruses), positive control samples infected by other viruses (PDV, PNRSV, ApMV, CVA, LChV-1, LChV-2, CNRMV, CRLV, CGRMV, PBNSPaV and ToRSV) and negative (water) control samples are shown in purple in panels a and b. Lanes 1—4, leaf samples from the T22 tree; 5–6, branch samples from the T22 tree in all three panels. Lane W, water control; H1 and H2, leaf samples from healthy flowering cherry trees H1 and H2; and L, 1 kb plus bp DNA ladder, are shown in panel (**C**). RNA extracts were used at a 10^−2^ dilution in both assays.

The optimized LAMP assay worked at the following reaction mix conditions: 2 µM of each inner prime (FIP and BIP), 0.2 µM of each outer primer (F3 and B3) and 1 µM of loop backward primer (LB) (Figure 6A). The melting temperatures of the LAMP assay had similar values in APLPV-infected samples of the T22 flowering cherry (84–84.5), while showing no peaks in healthy samples or the negative control (Figure 6B). Moreover, amplification signals were observed only in APLPV-positive samples, while no amplification was obtained in positive control samples infected by other viruses (PDV, PNRSV, ApMV, CVA, LChV-1, LChV-2, CNRMV, CRLV, CGRMV, PBNSPaV and ToRSV) or in negative control samples. The same RNA extracts (10^−2^ dilution) were also tested with the one-step RT-PCR assay developed in this study. The one-step RT-PCR assay detected APLPV in four out of six positive samples amplified by the LAMP assay, confirming its lower sensitivity (Figure 6C). Both assays were sensitive to detecting the virus in leaf samples (1–4) of the T22 flowering cherry, thus obtaining positive LAMP signals (from 4.88 to 7.71 min) and positive one-step RT-PCR results. Moreover, the LAMP assay was successful in APLPV detection in branch samples (5 and 6), while the one-step RT-PCR gave negative results. Although the same amount of RNA extract (0.5 ng total) was used in both assays, the slower LAMP detection of APLPV in branch samples (13.16–14.20 min) and the negative reaction by one-step RT-PCR may be related to the lower quality of RNA samples from the woody (hard) samples compared to leaf (soft) samples.

### 3.6. LAMP Assay in the Field

**Figure 7:**
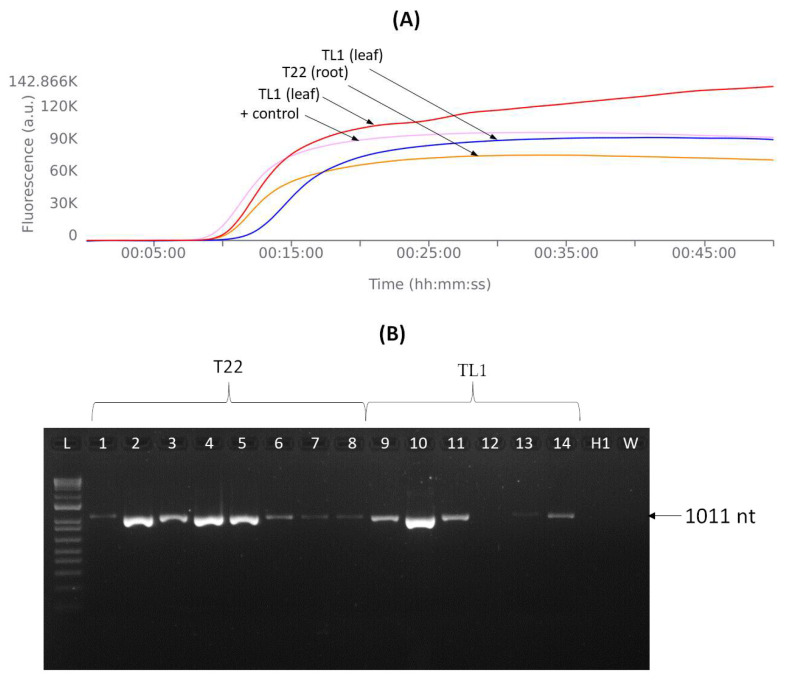
The in-field LAMP detection of APLPV and its comparison with specific one-step RT-PCR assays developed in this study. The results are visualized as follows: (**A**) LAMP; (**B**) agarose gel results of the one-step RT-PCR using APL_F1 x R primers. The amplification curves obtained from plant crude extracts in different extraction buffers correspond to the following samples in panel A: pink = positive control (RNA of APLPV PSer22IT isolate) with both, TET and ELISA, extraction buffers, red = leaf extract of flowering cherry TL1 (ELISA buffer), orange = root extract of flowering cherry T22 (ELISA buffer) and blue = leaf extract of flowering cherry TL1 (TET buffer). Panel B: Agarose gel electrophoresis of APLPV amplicons from naturally infected flowering cherry trees. Samples from different tissue materials of flowering cherry T22 (Lanes 1–8: roots) and flowering cherry TL1 (9–12: leaves, 13–14: branches); W, water control; H1, healthy flowering cherry control H1 grown at IPSP-CNR, Turin, that was not infected with Ilarviruses; L, 1 kb plus bp DNA ladder.

The LAMP assay was performed directly in the public garden where APLPV infection was previously found, and out of 20 tested flowering cherries, APLPLV was detected in 2 of them. The first positive sample corresponded to the root of the flowering cherry T22 from which the PSer22IT isolate originated. Surprisingly, the second positive sample was obtained from the leaves of an asymptomatic flowering cherry TL1 (Table 3 and Figure 7). APLPV infection in this tree was confirmed with specific one-step RT-PCR assays performed subsequently in the laboratory (Figure 7). LAMP detection times for APLPV were around 13 min in the T22 tree and 11 to 16 min in the TL1 tree depending on the extraction buffer. The TET buffer did not give a positive reaction with the APLPV-infected sample from the T22 tree, possibly due to the difficulty of obtaining crude extracts from roots, while it was slower in reacting with the APLPV-infected sample from the TL1 tree for 5 min. The ELISA extraction buffer proved to be a more suitable buffer and is suggested for use in future LAMP assays. Finally, the LAMP primers successfully detected APLPV in the field assay regardless of infection type: in the T22 flowering cherry (with a mixed APLPV + PNRSV + PDV infection) and in the L1 flowering cherry (with a single APLPV infection). The one-step RT-PCR assay was confirmed to be a less sensitive assay because it did not amplify APLPV from an RNA sample obtained from the leaf tissue of infected flowering cherry TL1. 

## 4. Discussion

APLPV was previously reported in Italy on flowering cherries from Verbania public gardens (Verbano-Cusio-Ossola province, Piedmont region) by PCR [4]. To the best of our knowledge, this study reports APLPV sequences in this plant host in Italy for the first time. Interestingly, we found again the presence of APLPV in the same public gardens in Verbania where the virus was reported in 2009. This may indicate the possibility of the natural transmission of the virus from tree to tree or that it was not found positive in the previous survey due to possible difficulties in detection—or it was an unsuitable season for the virus detection.

Some years before the first report of the virus on flowering cherry in the Piedmont region in 2009, APLPV was found on Japanese plum in a virus-infected stone fruit collection in Bari (Mediterranean Agronomic Institute, Valenzano—BA), and the scions from infected trees originated from Apulia and Sicily regions [3]. The virus was also found on naturally infected Japanese plum trees in Apulia [20]. Given that the virus vector is not known so far and that the virus was found in more geographies (Italy, Albania and Lebanon) and on three different hosts (Japanese plum, sweet cherry and flowering cherry), it suggests the virus introduction in Europe and the Mediterranean area through infected propagating material on more independent occasions.

Phylogenetic analyses of the APLPV *CP* and *MP* gene portions, including the PSer22IT isolate from this study and eleven reference GenBank isolates, were all isolates grouped into a single phylogroup indicating a low genetic variability in APLPV isolates from different parts of the world (eleven countries) and various hosts (six plant species). Similar low APLPV genetic variability was found in previous studies [7,10]. This confirmed that APLPV does not differentiate based on geographic origins or belong to different plant hosts. The low genetic variability of APLPV was also confirmed by the values of genetic diversity parameters obtained in our study and a previous study [10]. Taking into account the pairwise percent identity between APLPV sequences from Italy in both genes (*CP* and *MP*), it can be concluded that the sequences of the isolates from the same host are closer to each other compared to the isolates from other hosts. Thus, the isolates from Japanese plum have a higher genetic identity among them in both genes compared to the APLPV from the flowering cherry. This, together with the finding of APLPV on Japanese plum in southern Italy and flowering cherry in northern Italy, confirms the possibility of their independent introduction in Italy. These results are in agreement with studies on other Ilarviruses [33,34]. 

In this study, two RT-PCR assays were developed for the specific detection of APLPV that enable the amplification of the full *CP* and *MP* genes and their external regions. In addition to diagnostic purposes, these RT-PCR assays using the designed primers can be useful for the sequencing and molecular characterization of the virus since they amplify the regions longer than 1000 nt. The primers developed in previous studies amplified shorter APLPV products (600 nt or less) of the gene portions that are more suitable for detection than for phylogenetic analyzes of the virus [9,19,22].

In the present work, we developed a rapid and sensitive RT-LAMP assay for the specific detection of APLPV in flowering cherry. As far as we know, no molecular isothermal amplification method is available for APLPV detection in plants. The developed RT-LAMP assay in our study is sensitive since it has the potential to detect APLPV in RNA extracts of flowering cherry leaf samples up to 10^−5^ dilutions, which is in line with other LAMP assays used in the detection of plant RNA viruses [35,36]. 

The new LAMP assay was shown to be more sensitive in APLPV detection in flowering cherry compared to the specific one-step RT-PCR assay. It was able to detect the virus up to 10^−5^ RNA dilution, while the one-step RT-PCR detected APLPV only to 10^−3^ RNA dilution. This could be explained by the greater resistance of *Bst* DNA polymerase employed in LAMP, which is less sensitive to inhibitors and has higher specific activity, catalytic constant, and processivity in comparison to *Taq* DNA polymerase [37,38]. Furthermore, we added AMV reverse transcriptase to the developed LAMP reaction mix. Even though the GspSSD LF DNA polymerase in the isothermal ISO-004 mix has native reverse transcriptase activity, AMV reverse transcriptase was additionally added to the reaction mix to improve assay efficacy, confirming the results of the previous LAMP studies [39].

The LAMP assay developed in this study proved to also be rapid because it allows virus detection within 20 min from the start of the assay. It is also specific considering that it did not give non-specific reactions with other stone fruit viruses and healthy samples. 

Finally, it gave the possibility to be performed directly in the field without the need for the extraction of RNA from samples in the laboratory, requiring only the quick in-field preparation of crude plant extracts. The most effective plant crude extract preparation was obtained with the ELISA extraction buffer, which also performed successfully in the LAMP detection of Flavescence Dorée phytoplasma in grape vine host and *Scaphoideus titanus* vector [31,40]. This may be related to the salt concentration, pH value and reagents contained in the ELISA extraction buffer, which are similar to those in which *Bst* polymerase has optimal activity [38]. The developed LAMP method has great potential for large-scale monitoring in stone fruit orchards in the future and the possibility to be performed directly in the field, as already reported with other important plant pathogens [31,32,41].

Considering that it is a plant quarantine pathogen, further studies are necessary to estimate its geographic spread in the territory, paying particular attention to surrounding gardens and vegetatively propagated flowering cherries in ornamental nurseries. For this, the developed LAMP assay will certainly be useful because it will help evaluate new infections, monitor the development of the virus within different seasons and enable timely and necessary management practices.

In conclusion, this study reported the first LAMP APLPV-specific assay and first molecular characterization of APLPV from flowering cherry in Italy and confirmed its presence in the same territory in Italy where it was found 15 years ago. This indicates the need for deeper research into the epidemiology of this virus and the way of its natural transmission using early and rapid molecular diagnostics.

## Figures and Tables

**Table 1:** List of primers used for APLPV detection by LAMP.

Number	Name	Sequence (5′-3′)	Gene	Primer Position (nt) *	Final Concentration in LAMP Reaction
**1**	F3_AMP	TCTATCTAAGGCTGTGCTAG	Movement protein	357–376	0.25 μM
	B3_AMP	TCATAGGAGGTCTCTCCTTC		525–544	0.25 μM
	FIP_AMP	CGGAGACTTCATAGGCGAGATTTAA-GATGACGAATTGGACTAGGT		418–442 (F1c) + 378–397 (F2)	2.5 μM
	BIP_AMP	TCAACAAATAGGGAGATTCCGGA-TCGTAGATCTGCTTGGAAC		462–484 (B1c) + 506–524 (B2)	2.5 μM
	LB_AMP	TACTTTGGGCAGATGTCCCA		485–504	1.25 μM
**2**	F3_ACP	CTCGCGGTGAGATTGAAACT	Coat protein	89–108	0.25 μM
	B3_ACP	TACTGACCAGCGGTCGTC		261–278	0.25 μM
	FIP_ACP	TCCCACTCCGTGCGTATGGA-TATAACACCTGCGCGAAGTG		160–179 (F1c) + (F2) 120–139	2.5 μM
	BIP_ACP	TGGTAGGTCCTTCTGTGGGTGC-AGAGGCAACTGCCTCTCC		182–203 (B1c) + 241–258 (B2)	2.5 μM
	LF_ACP	CGAGGAACTGGTGGACCAC		140–158	1.25 μM
	LB_ACP	CCGTTTAATTCAGGGGCCTCACAA		204–227	1.25 μM

* The numbers at the primer position refer to the beginning of the CDS of the gene.

**Table 2:** Molecular parameters of APLPV isolates obtained from complete *CP* and partial *MP* genes.

Polymorphism Parameter	CP	MP
Number of isolates	14	13
Analyzed region	1–654	1–802
Number of polymorphic sites, S	37	60
Number of mutations, Eta	40	62
Nucleotide diversity, π	0.01406	0.01613
θ (S)	0.02120	0.02531
θ (π)	0.01433	0.01648
ds	0.04196	0.05152
dNS	0.00387	0.00568
Tajima’s D	−1.56786	−1.58895
Fu and Li’s D	−1.86773	−1.86663
Fu and Li’s F	−2.05034	−2.05147

**Table 3:** In-field detection of APLPV using crude extracts of flowering cherry plants by LAMP.

Flowering Cherry Trees	Samples	LAMP Tp (Min) *	
		ELISA Buffer	TET Buffer
T22	Root	13.02 ± 0.76	nd
TL1	Leaf	10.80 ± 1.12	16.35 ± 3.00
TL2-TL19 **	Leaf	nd ***	nd
Positive control	Leaf	10.50	10.49
Negative control	Leaf	nd	nd

* Tp = Time to positive; ** corresponds to the remaining 18 tested flowering cherry trees in the field; *** nd = not determined. RNA containing APLPV PSer22IT isolate and RNA extracted from leaves of healthy flowering cherry (H1), which was not infected with Ilarviruses, were used as positive and negative controls.

## Data Availability

All the data are available in the manuscript.

## Supplementary Materials

The following supporting information can be downloaded at https://www.mdpi.com/article/10.3390/v16101572/s1: Figure S1. Agarose gel electrophoresis of APLPV-specific one-step RT-PCR assay as described by Sánchez-Navarro et al. (2005) to amplify virus partial coat protein (CP) gene (563 bp); Figure S2. Graphical overview of pairwise nucleotide identity (%) of nucleotides of the sequenced genes between APLPV isolates originating from Italy and other countries using the SDTv1.0 program: (A) coat protein gene and (B) movement protein gene; Figure S3. Real-time LAMP results for the detection of APLPV in flowering cherry T22 with the set of primers designed in the MP and the CP genes.
