# Supplementary material for: Development of Loop-Mediated Isothermal Amplification (LAMP) Assay for In-Field Detection of American Plum Line Pattern Virus
Source: Viruses. 2024 Oct 5;16(10):1572. doi: 10.3390/v16101572 (PMC11512406; doi:10.3390/v16101572)
Supplement: Supplementary file 1 [file viruses-16-01572-s001.zip › viruses-3237751-supplementary.pdf]

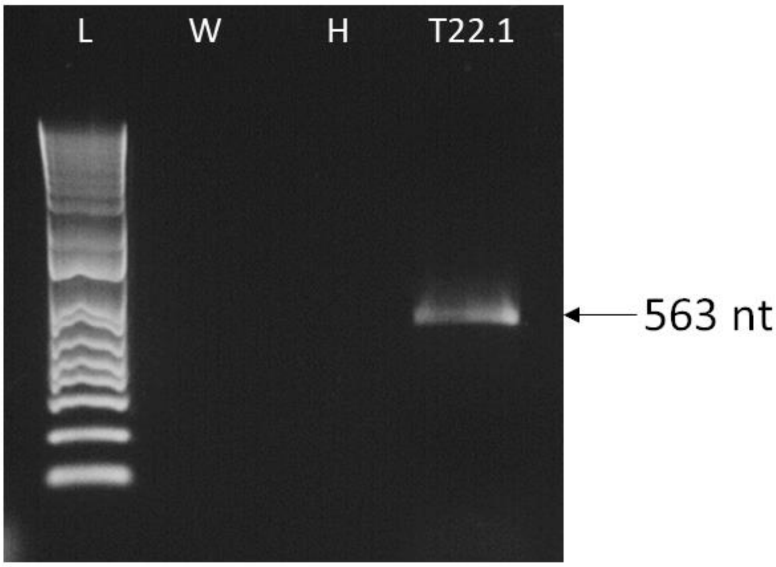

Figure S1. Agarose gel electrophoresis of APLPV-specific one-step RT-PCR assay as described by Sánchez-Navarro et al. (2005) to amplify the virus partial coat protein (*CP*) gene (563 bp). L, 1kb plus bp DNA ladder; W, water control; H, healthy flowering cherry control H grown at IPSP-CNR; T22.1 – flowering cherry T22.

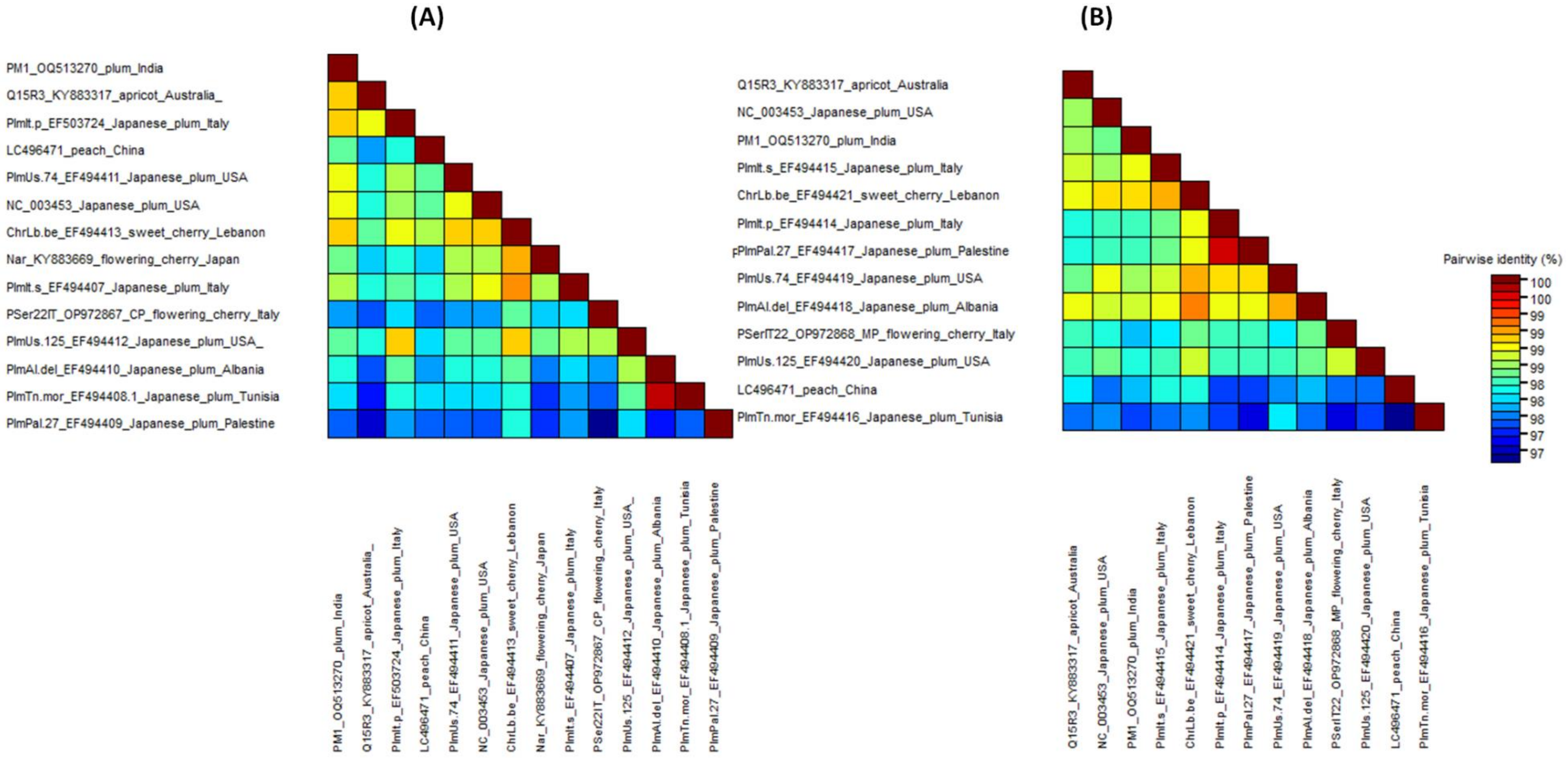

Figure S2. Graphical overview of pairwise nucleotide identity (%) of nucleotides of the sequenced genes between APLPV isolates originating from Italy and other countries using the SDTv1.0 program. (A) coat protein gene and (B) movement protein gene. Each colored cell indicates a percentage of the identity score between the two sequences.

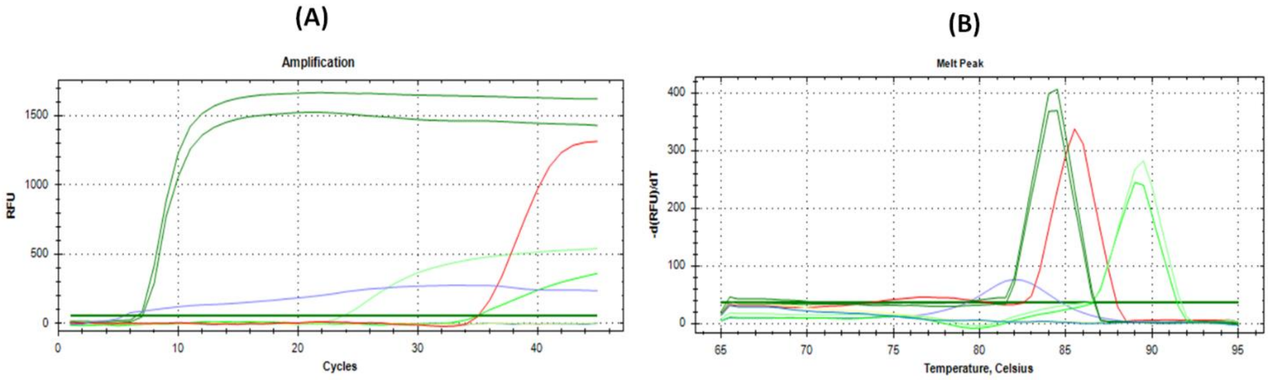

Figure S3. Real-time LAMP results for the detection of APLPV in flowering cherry T22 with the set of primers designed in the *MP* and the *CP* genes. The results are visualized as: A) amplification results; B) melting curve results. Infected samples are indicated by dark green colour (the first set of primers) and light green colour (the second set of primers), healthy control is shown by dark blue colour (the first set of primers) and light blue colour (the second set of primers), and water control is indicated by yellow colour (the first set of primers) and red colour (the second set of primers). Flowering cherry (H1), which was not infected with Ilarviruses was used as a healthy control.
